# Factors associated with patients’ choice of physician in the Korean population: Database analyses of a tertiary hospital

**DOI:** 10.1371/journal.pone.0190472

**Published:** 2018-01-02

**Authors:** Kidong Kim, Soyeon Ahn, Banghyun Lee, Kibeom Lee, Sooyoung Yoo, Kyogu Lee, Dong Hoon Suh, Jae Hong No, Yong Beom Kim

**Affiliations:** 1 Department of Obstetrics and Gynecology, Seoul National University Bundang Hospital, Seongnam-Si, Gyeonggi-Do, Republic of Korea; 2 Divison of Statistics, Medical Research Collaborating Center, Seoul National University Bundang Hospital, Seongnam-Si, Gyeonggi-Do, Republic of Korea; 3 Department of Obstetrics and Gynecology, Hallym University Kangdong Sacred Heart Hospital, Seoul, Republic of Korea; 4 Program in Digital Contents & Information Studies, Graduate School of Convergence, Science and Technology, Seoul National University, Seoul, Republic of Korea; 5 Center for Medical Informatics, Seoul National University Bundang Hospital, Seongnam-Si, Gyeonggi-Do, Republic of Korea; 6 Seoul National University, School of Medicine, Seoul, Republic of Korea; Indiana University School of Medicine, UNITED STATES

## Abstract

This study aimed to determine the factors influencing patients’ choice of physician at the first visit through database analysis of a tertiary hospital in South Korea. We collected data on the first treatments performed by physicians who had treated patients for at least 3 consecutive years over 10 years (from 2003 to 2012) from the database of Seoul National University’s affiliated tertiary hospital. Ultimately, we obtained data on 524,012 first treatments of 319,004 patients performed by 115 physicians. Variables including physicians’ age and medical school and patients’ age were evaluated as influencing factors for the number of first treatments performed by each physician in each year using a Poisson regression through generalized estimating equations with a log link. The number of first treatments decreased over the study period. Notably, the relative risk for first treatments was lower among older physicians than among younger physicians (relative risk 0.96; 95% confidence interval 0.95 to 0.98). Physicians graduating from Seoul National University (SNU) also had a higher risk for performing first treatments than did those not from SNU (relative risk 1.58; 95% confidence interval 1.18 to 2.10). Finally, relative risk was also higher among older patients than among younger patients (relative risk 1.03; 95% confidence interval 1.01 to 1.04). This study systematically demonstrated that physicians’ age, whether the physician graduated from the highest-quality university, and patients’ age all related to patients’ choice of physician at the first visit in a tertiary university hospital. These findings might be due to Korean cultural factors.

## Introduction

When patients must select a care provider due to new health events, many rely passively on primary physician referral, distance to the care provider, or previous experiences. Only a small number of patients (10–13%) tend to actively choose a care provider, and these may include highly educated and young patients, patients with high income, and patients having a bad relationship with their previous provider, although various studies have reported conflicting results [1,2]. Meanwhile, a minority of patients (e.g., 14%) have no opportunity to choose a care provider because of need for urgent care, difficulty, or illogicality of changing care already begun [2].

Patients’ choices of medical care are believed to be determined by complex interaction between the characteristics of the patient and provider. Usually, patients aim to obtain high-quality care while minimizing costs. However, because they often lack information in this regard, most patients cannot make a completely rational choice. Therefore, they tend to choose their physician based on providers’ characteristics from among many in the hospital that treat the same condition [1].

Some studies have examined how patients’ choice is influenced by characteristics of the physician, such as age, gender, medical school/residency, specialization, availability, communication skill, ability to offer treatment continuity, and waiting time [3–9]. Physicians’ age and gender are perhaps the most commonly investigated influencing factors [8,9]. Whether patients received high-quality medical treatment is also an important influencing factor [5,10]. Moreover, patients’ choices are influenced by recommendations from significant others, such as family or friends [7,11]. A patient’s own characteristics, such as gender, are reported to have rather weak associations with their chosen physicians’ characteristics [7]. However, few studies have systematically evaluated the basis on which patients choose their physician [1].

In previous studies, the demographic characteristics of physicians were considered weaker factors associated with patients’ choice compared with factors such as professional expertise [1,10]. For example, a study involving administration of a 23-item survey to 636 community residents in the USA showed that participants rated professionally relevant factors and management practices, such as “whether the doctor is board certified” and “time to get an appointment,” as more important for patients’ choice than demographic characteristics, such as “race, age, and gender” [10]. However, these characteristics might differ by country or region, among other situations. Additionally, culture is described as the framework that guides and binds life practices [12,13]. Therefore, it is possible that cultural factors influence the weight given by patients to certain physicians’ characteristics [12,13].

According to previous studies, cultural factors based on racial/ethnic disparities may contribute to patients’ choice of physician, as follows: White patients are more likely than Black and Hispanic patients to select their surgeon and hospital based on reputation [14]; Pakistani or Indian patients prefer Asian primary physicians, and Caucasian patients prefer Caucasian primary physicians [15]. Moreover, today, many patients in East Asian countries still have the choice to see a doctor who practices Western or Traditional and Complementary (or Oriental) medicine for their disease, suggesting that East Asians might differ in this respect from the rest of the world. In other words, East Asians continue to select traditional and complementary medicine, suggesting their conservative predisposition, although there are great advances in Western medicine. Therefore, East Asians might also have a tendency toward conservatism in selection of a physician, such as a preference for old and male physicians. However, no studies have yet systematically investigated the cultural factors specific to East Asian countries that relate to patients’ choice of physician; furthermore, no studies have yet investigated the determinants of patients’ choice of physician in South Korea specifically. Therefore, in this study, we investigated the influencing factors associated with patients’ choice of physicians at the first visit through analyses of a database in a Korean tertiary hospital.

## Materials and methods

This exploratory study was approved by the Institutional Review Board of Seoul National University Bundang Hospital (No. B-1310/224-104) on November 1, 2013. Informed consent was waived. Our study included all patients who had received their first treatment at Seoul National University Bundang Hospital between April 1, 2003, and December 31, 2012. Patients’ information was collected from the hospital’s database (medical records). We included data only from patients who had received their first treatment from a physician who had treated patients for at least 3 consecutive years. We decided not to include data on how many physicians performed a first treatment for a small number of patients over a short period because such data may have complicated the analyses without significantly contributing to the outcomes.

In total, we obtained data from 319,004 patients and 115 physicians. The events for which these patients received their first treatment totaled 524,012. When permitting for duplication of physicians according to the study years, data for 674 physicians were collected.

The independent variables collected were physicians’ age and gender, proportion of male patients for each physician, the medical schools that physicians attended, patients’ age and gender, and hospital department in which the event was treated. Based on whether they had attended medical school at Seoul National University (SNU), physicians were classified into the categories “from SNU” and “not from SNU.” SNU’s medical school has been considered the highest-quality medical school in Korea. We also noted the year of the first treatment, which we adjusted for in our analyses because the hospital has grown in size annually since it opened in 2003.

The dependent variable was the number of first treatments by each physician in each year. We assumed that the number of first treatments provided by physicians was directly linked with how many patients preferred them at the first visit. Patients tend to seek treatment from the same physician repeatedly after choosing them, for continuity of treatment. However, other factors, such as trust in physicians, might influence patient choice of physician in subsequent treatments [16]. Therefore, we analyzed the number of first treatments to clearly evaluate the influencing factors of patients’ choice of physicians with minimal confounding.

Gynecologists, a statistician, and experts in digital content programming and information studies participated in this study from the initial planning stages to the systematic analysis of factors influencing first treatment. All of them interacted in identification of the study’s purpose, establishment of the design, data extraction from the database, statistical analyses, and interpretation of results.

### Statistics

Data for continuous variables are expressed as means ± standard deviations, while those for categorical variables are expressed as numbers and percentages. We first explored the effects of patient characteristics on physician characteristics using concordance analysis and mixed-effects models. Mixed-effects models were able to infer how a patient’s characteristics explained the corresponding physician’s characteristics by physician-specific parameterization. We then investigated the association between patients and physicians using the yearly number of first treatments per physician as the outcome variable. Statistical analyses were repeated using physicians’ age and the median age of physicians to obtain more accurate results. To obtain population-averaged estimates, a generalized estimating equation with a Poisson regression using a log link was implemented to account for a physician clustering effect. To evaluate whether specific variables were independent risk factors for patients’ choice of physician, multivariable regression analyses were performed. R 3.2.0 was used for all statistical analyses.

## Results

At the baseline (i.e., demographic characteristics of patients and physicians) and first treatment, the proportion of male patients was smaller than that of female patients, while the proportion of male physicians was larger than that of female physicians. Moreover, the proportion of physicians graduating from SNU was much larger than that of physicians not from SNU, and this proportion increased further at first treatment compared to the baseline. Additionally, at first treatment, the mean age of male patients was similar to that of female patients, whereas the mean age of male physicians was older than that of female physicians. The department in which patients most frequently received their first treatment was neurology, followed in order by family medicine, orthopedics, internal medicine (division of pulmonology), general surgery, and others (Table 1). The distributions of patients and physicians according to study year were similar. However, the number of patients increased rapidly until 2005 and then more slowly until 2009. After 2009, a rapid and continuous decrease in patients was seen. The number of physicians increased until 2010, after which their number decreased continuously (data not shown).

**Table 1:** Characteristics of patients and physicians according to baseline features and events at first treatment.

Characteristic	Patients	Physicians	Event at first treatment	
	(n = 319,004)	(n = 115)	(n = 524,012)	
			**Patients**	**Physicians**
			(n = 524,012)	(n = 524,012)
**Age, years (mean ± SD)**				
**Female**			52.7 ± 15.6	36.5 ± 4.1
**Male**			53.6 ± 16.0	43.1 ± 7.3
**Gender, n (%)**				
**Female**	178,043 (55.8)	25 (21.7)	295,288 (56.4)	93,915 (17.9)
**Male**	140,961 (44.2)	90 (78.3)	228,724 (43.6)	430,097 (82.1)
**Medical school attended by physician, n (%)**				
**From SNU**		89 (77.4)		466,249 (89.0)
**Not from SNU**		26 (22.6)		57,763 (11.0)
**Specialization/hospital department, n (%)**				
**Neurology**		11 (9.6)		65,505 (12.5)
**Family medicine**		4 (3.5)		38,880 (7.4)
**Orthopedics**		6 (5.2)		34,079 (6.5)
**Internal medicine (division of pulmonology)**		5 (4.3)		33,285 (6.4)
**General surgery**		7 (6.1)		31,597 (6.0)
**Others**				

SNU: Seoul National University

Concordance analysis and mixed-effects models showed that older patients were more likely to choose an older physician. The proportion of male patients opting for female physicians was similar to that of male physicians (Tables 2 and 3, and S1 Table).

**Table 2:** Results of concordance analysis and mixed-effects model between patients and physicians according to age.

	Age of physician, years^a^			
	29 to 39 (n = 264)	40 to 49 (n = 259)	50 to 59 (n = 118)	60 to 75 (n = 33)
**Age of patient, years (median [IQR])**	51.6 [47.4, 55.3]	53.9[47, 58.6]	51.3 [42, 57.7]	53.9 [41.7, 55.1]
**Patient age (coefficient)**^b^	0.0074 (0.0070, 0.0077)			

^a^The number of physicians indicates the number without adjustment for duplication of physicians during the study period.

^b^Results of a univariable linear mixed-effects model expressed in estimate and 95% confidence interval.

IQR: interquartile range

**Table 3:** Results of concordance analysis and mixed-effects model between patients and physicians according to gender.

	Physician’s gender^a^	
	Female (n = 137)	Male (n = 537)
**Proportion of male patients, % (median [IQR])**	47.9 [34.8, 54.8]	43.1 [36.2, 50.7]
**Male patients (odds ratio)**^b^	1 (reference)	1.011 (0.007, 146.89)

^a^The number of physicians indicates the number without adjustment for duplication of physicians during the study period.

^b^Results of a univariable mixed-effects logistic regression model expressed in estimate and 95% confidence interval. 10% of patients were randomly selected owing to a computational burden from a large sample size.

IQR: interquartile range

**Fig 1:**
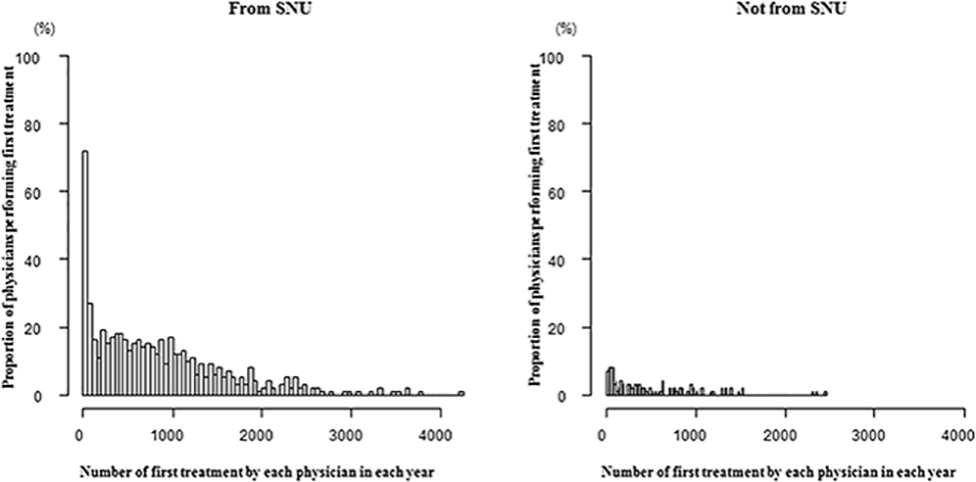
Number of first treatments performed by each physician according to the medical school attended. Proportion of physicians was not adjusted for duplication of physicians over the study period. SNU: Seoul National University.

In the analysis including physicians’ age, the number of first treatments decreased over time. The relative risk of first treatment was lower in older physicians than in younger ones. However, being a male physician and the proportion of male patients for each physician showed no associations with the number of first treatments. Interestingly, the relative risk of first treatment was greater among physicians from SNU than physicians not from SNU (Fig 1). The relative risk of first treatment was also higher among older patients compared with younger patients. When using the anesthesiology department as a reference, because “anesthesiology” has “A” as its initial letter, in comparing differences among hospital departments, the relative risk of first treatment was higher for family medicine, dermatology, health screening and promotion center, urology, and others (Table 4). Notably, when conducting all analyses with the median physician age, the outcomes were similar to those of analyses including the raw ages (data not shown).

**Table 4:** Factors associated with patients’ choice of physician based on multivariable regression analyses.

	RR (95% CI)^a^
**Year of first treatment**	0.95 (0.93 to 0.97)
**Age of physician (years)**	0.96 (0.95 to 0.98)
**Gender of physician (male)**	1.22 (0.99 to 1.5)
**Proportion of male patients to each physician**	0.69 (0.29 to 1.61)
**Medical school attended by physician (from SNU)**	1.58 (1.18 to 2.10)
**Age of patient (mean, years)**	1.03 (1.01 to 1.04)
**Specialization/hospital department**	
**Family medicine**	10.11 (3.59 to 28.44)
**Dermatology**	6.58 (2.43 to 17.84)
**Health screening and promotion center**	5.76 (2.15 to 15.43)
**Urology**	5.5 (1.64 to 18.37)
**Others**	

RR: relative risk; CI: confidence interval; SNU: Seoul National University

^a^A generalized estimating equation was implemented.

## Discussion

In this exploratory study, we analyzed the database of a tertiary university hospital to identify factors associated with patients’ choice of physicians at the first visit in the South Korean population. Our results showed that the physicians’ age and medical school (i.e., status as a graduate of the highest-quality university) as well as patients’ age were all significant influencing factors for patients’ choice of physician at the first visit. However, the physicians’ gender and proportion of male patients treated by each physician were not influencing factors. Furthermore, we noted that the relative risk of patients receiving first treatment was dependent on hospital departments.

Interestingly, there was a significant association between the number of first treatments and younger physicians. This finding may suggest that patients consider current evidence-based standards of care more important than the physician’s experience when they visit a tertiary hospital. However, it should be noted that in the SNU hospital, patients may be more frequently allocated to younger physicians for first treatment because older physicians usually have more patients, making it more difficult for them to fit in new appointments. Therefore, it is possible that our results reflect this situation. In other words, younger physicians performing more first treatments might suggest a lack of choice on the part of the patients. However, when we analyzed physicians over 40 years old (i.e., the median age of the physicians included in the study), the relative risk of first treatment was not different from that in analysis of all physicians (relative risk 0.96; 95% confidence interval 0.95 to 0.98). Physicians over 40 years old have a large number of established patients, and are less influenced by new patients. Therefore, higher rates of first treatment among younger physicians in light of all these findings may show patient preference for younger physicians.

As noted above, one of the main findings was that physicians graduating from SNU had a higher relative risk of performing first treatments compared to those not from SNU, suggesting that patients more frequently choose the former category. However, there is still potential for bias contributing to this association: first, patients had a higher chance of choosing the former group of physicians because a larger proportion of the physicians in the SNU hospital also attended SNU; second, patients may have been referred to these physicians by other physicians who had attended the same medical school. The results regarding association between older patient age and first treatment may be explained by the general expectation that older patients develop disease more frequently than do younger patients.

In South Korea, patients can choose to visit clinics or hospitals for a physician consultation without a referral slip. This system has led to a competitive relationship between clinics and hospitals. In contrast, in Western countries, clinics generally provide care for outpatients, whereas patients generally must receive a referral letter from their primary physicians before going to a hospital. In this sense, clinics and hospitals have a collaborative relationship [17]. In the present study, the fact that family medicine physicians were the most likely to perform first treatments is reflective of South Korea’s primary care system, wherein many patients visit tertiary hospitals to receive primary care. Moreover, the high frequency of first treatment in the health screening and promotion center also shows a trend of medicine in South Korea, in which tertiary hospitals expand their role in primary screening of disease.

Some previous studies have reported that patients’ demographic characteristics such as gender and marital status do not significantly affect their choice of medical provider and have little influence on the perceived significance of primary care physicians’ characteristics (e.g., their gender, marital status and race) [10,18]. The results of our study showed no relationship between patients’ gender and physicians’ gender, supporting previous research [10], whereas patients’ age showed a positive correlation with physicians’ age. In support of previous studies [10,18], our study indicated that patients’ gender was not a factor influencing association between first treatment and physicians. Moreover, physicians’ gender was also not associated with the risk ratio of first treatment, which supports the findings of a previous study that gender preferences are less important in physician selection (even in selection of obstetrician-gynecologists) [9].

Additionally, in our study, there might be possible confounding factors that were not included in the adjustment for our analysis. However, the following issues with relation to confounding factors need to be considered. Insurance coverage is no longer an influencing factor for selecting medical services because of a wide range of national health services in Korea. The majority of Koreans are of the same race. Therefore, Korea does not permit the consideration of race although the number of migrants is slowly increasing. Moreover, including every patient across all departments may limit the selection of more confounding factors, such as disease-specific or age-specific factors.

At the start of this study, we supposed that the Korean (East Asian) population would prefer older and male physicians and physicians graduated from the highest-quality university at the first visit. Consequently, preference for physicians who graduated from the highest-quality university show a population disposition to deem reputation one of the most important considerations in choice of physician. Preference for younger physicians, and no preference for physicians’ gender, may show changes that occurred over time in South Korea.

In the present study, we systematically evaluated the influence of patient and physician characteristics on patients’ choice of physician at the first visit using a large sample through analysis of the database of a tertiary hospital. However, as noted above, some bias might have influenced our results. Even considering this potential bias, the operating system of the hospital might be modified to balance treatment across all physicians irrespective of their age and whether they graduated from SNU, and to encourage younger patients to visit. Additionally, our results well demonstrated the influence of cultural factors inherent in the South Korean population on patients’ choice of physician was well demonstrated in our results. Further hospital database analyses using various statistical techniques might be used to effectively increase the range of our results and reduce possible bias in the future.

## Supporting information

S1 Table Concordance analysis between patients and physicians according to age and gender: stratification based on the number of first visits. The number of physicians indicates the number without adjustment for duplication of physicians during the study period. IQR: interquartile range.
(DOCX)
