# Supplementary material for: Factors associated with patients’ choice of physician in the Korean population: Database analyses of a tertiary hospital
Source: PLoS One. 2018 Jan 2;13(1):e0190472. doi: 10.1371/journal.pone.0190472 (PMC5749849; doi:10.1371/journal.pone.0190472)
Supplement: S1 Table — The number of physicians indicates the number without adjustment for duplication of physicians during the study period. IQR: interquartile range. (DOCX) [file pone.0190472.s001.docx]

**S1 Table. Concordance** **Analysis between Patients and Physicians According to Age and Gender: Stratification based on the number of First Visits.**

|  |  | **The number of first visits per physician** | | | |
| --- | --- | --- | --- | --- | --- |
|  |  | 0 to 192 (n = 169) | 193 to 612 (n = 168) | 613 to 1140 (n = 170) | 1141 to 4300 (n = 167) |
|  | **Age ranges of physicians** |  |  |  |  |
| **Age of patients, years (median [IQR])** | 29 to 39  40 to 49  50 to 59  60 to 75 | 51.9 (36.2, 55.9)  51.2 (40, 56.9)  40.3 (33.5, 44.8)  32.2 (25.6, 50.8) | 51.3 (46.7, 54.9)  52.6 (44.9, 59.4)  52.2 (47.7, 57)  54.8 (54, 55.9) | 51.4 (48.2, 54.4)  55.6 (47.3, 59.9)  53.1 (47.1, 65.1)  54.8 (54.2, 57.3) | 51.8 (48.5, 55.8)  54.5 (51.8, 56.6)  54 (50.6, 59.5)  62.1 (62.1, 62.1) |
|  | **Physicians’ gender** |  |  |  |  |
| **Proportion of male patients (median [IQR])** | Female  Male | 37.3 (32.6, 50.7)  40.3 (23.6, 50) | 50.4 (39, 53.4)  42.9 (36.1, 51.6) | 52.6 (39.2, 57.5)  43.8 (38.6, 48.8) | 51.1 (26.3, 56.8)  44.4 (38.3, 52.2) |

The number of physicians indicates the number without adjustment for duplication of physicians during the study period.

IQR: interquartile range
